# Synthesis and Characterization of Polyethylene Glycol-Grafted Photoreactive Polyethylene Glycols for Antibiofouling Applications

**DOI:** 10.3390/polym15010184

**Published:** 2022-12-30

**Authors:** Mahmoud H. Othman, Yoshihiro Ito, Jun Akimoto

**Affiliations:** 1 Emergent Bioengineering Materials Research Team, RIKEN Center for Emergent Matter Science, 2-1 Hirosawa, Wako 351-0198, Saitama, Japan; 2 Department of Biological Sciences, Graduate School of Science, Tokyo Metropolitan University, 1-1 Minami-Osawa, Hachioji 192-0397, Tokyo, Japan; 3 Nano Medical Engineering Laboratory, RIKEN Cluster for Pioneering Research, 2-1 Hirosawa, Wako 351-0198, Saitama, Japan

**Keywords:** photoreactive, azidophenyl, polyethylene glycol, photoimmobilization, cell adhesion, protein adsorption, antibiofouling

## Abstract

Notably, antibiofouling is an important and predominant technique adopted to improve the surfaces of biomaterials. In this study, polyethylene glycol-grafted polyethylene glycols bearing azidophenyl groups were synthesized and immobilized on polystyrene surfaces via photoirradiation. The prepared polymers were found to be highly soluble in water, and photoimmobilization with fluorescent proteins was confirmed based on micropatterning using a photomask. These polymers suppressed nonspecific interactions between proteins and cells on the substrate. Considering that photoimmobilization can be adopted for the covalent bond modification of various surfaces, the developed water-soluble and highly antibiofouling polymers appear to be useful in biomaterial preparation.

## 1. Introduction

Typically, surface functionalization can facilitate the control of interactions between target materials while simultaneously suppressing nonspecific interactions with the surfaces. In particular, non-biofouling surfaces resist the adsorption of proteins and/or cell adhesion. This is because proteins generally have a strong tendency to adsorb on almost all surfaces, leading to cell adhesion. Therefore, surfaces that resist protein adsorption also resist cell adhesion [1,2].

There have been many recent advances in the construction of anti-biofouling surfaces for preventing nonspecific binding (fouling) in biomedical fields. For example, a microelectrode coated with a polyethylene glycols (PEG) methacrylate polymer (anti-biofouling layer) was developed to facilitate its implantation into the rat brain for electrophysiological recordings [3,4].

A nanosensor coated with a PEG–lipid was shown to minimize protein fouling and to prolong nanoparticle blood circulation for the monitoring of histamines [5,6]. Nanoparticles coated with a PEG polymer network (subsequently an antifouling shell) are capable of the in vivo detection of hydrogen peroxide (H_2_O_2_) that indicates many pathological processes including chronic diseases such as cancer. PEG was shown to endow the particles with good fouling resistance, water solubility, and biocompatibility [7].

The development of anti-biofouling membranes for inhibiting membrane biological contamination in the field of water purification was also established. The coated membranes were shown to inhibit the initial attachment or deposition of biological macromolecules/microorganisms and to prevent the subsequent growth and multiplication of adhered microbes [8].

To date, various polymers have been used to suppress nonspecific interactions in cells; these include polyethylene glycol (PEG) [9,10,11,12,13], zwitterionic species [14,15,16,17], peptides [18], polypeptides [19], and glycocalyx [20]. These polymers are highly hydrophilic and flexible, and they exhibit nonadherent and nonbiofouling properties. Furthermore, the immobilization of these polymers on antibiofouling surfaces has been achieved with various methods, including self-assembly [21], electro-grafting [22], and polymerization; in this regard, atom transfer radical polymerization [23] and reversible addition–fragment chain transfer polymerization have also recently been employed [24]. Among these methods, photoimmobilization is interesting because it enables the covalent immobilization of the antibiofouling layer [25]. Therefore, some types of photoimmobilizable (reactive) polymers containing PEG [26,27], zwitterions (such as phosphorylcholine) [28,29,30], sulfobetaine [31], carbobetaine [32], pullulan [33], sulfated hyaluronic acid [34], polyvinyl alcohol [35] and polyacrylate derivatives [36] have been used for the preparation of antibiofouling surfaces.

Among the photoreactive polymers, photoreactive antifouling acrylic polymers grafted with PEG have been used as microarray matrixes [37,38,39,40,41,42,43] and are now commercially employed in allergy diagnosis (DropScreen^R^) [44,45]. In a previous study, photoreactive PEG was synthesized via the copolymerization of methacrylate-PEG and acryloyl 4-azidobenzene. The photoreactive polymer was photoimmobilized on plastic, glass, and titanium surfaces for antibiofouling surface modification [26]. This immobilization technique is indeed extremely useful for surface antibiofouling modification due to its convenience. The modified surface significantly reduces interactions with proteins and can be used for protein immobilization via photo-induced crosslinking [26].

However, owing to the low hydrophilicity of the polymethacrylate backbone, the water solubility of these polymers is relatively low and further decreases following an increase in the number of azidophenyl groups [13,27]. To enhance water solubility, we synthesized a PEG-based photoreactive polymer composed of ethylene oxide and azidophenyl-coupled ethylene oxide. However, its water solubility was still low. Therefore, in this study, co-immobilization with PEG-carrying ethylene oxide was performed to enhance its water solubility and photoreactivity, and the resulting antibiofouling properties were investigated.

## 2. Materials and Methods

### 2.1. Materials

4(Glycidyloxymethyl)azidobenzene (AzPheEO) was prepared according to our previous report [21]. Methoxy-PEG epoxide (mPEG-EPO, Mn = 350 and 750) was purchased from Biochempeg Scientific (Boston, MA, USA). Tetrabutylammonium bromide (TBAB), ethanol, methanol, *n*-hexane, and *N,N*-dimethylformamide were purchased from FUJIFILM Wako Pure Chemical (Osaka, Japan). Ethylene oxide (EO, 1.0 mmol/L toluene solution), 4-azidobenzoic acid, lithium bromide, and triisobutyl aluminum (i-Bu_3_Al, 1.0 mol/L toluene solution) were purchased from Tokyo Chemical Industry (Tokyo, Japan). Tissue culture polystyrene dishes were purchased from Iwaki (Tokyo, Japan). Alexa488-conjugated immunoglobulin G (Alexa488–IgG), fetal bovine serum (FBS), and trypsin were purchased from Thermo Fisher Scientific (Waltham, MA, USA). A penicillin–streptomycin solution was purchased from Nacalai Tesque (Kyoto, Japan).

### 2.2. Copolymerization of EO, AzPheEO, and mPEG-EPO

**Scheme 1:**
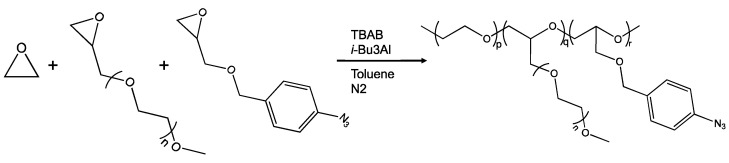
Preparation of photoreactive polyethylene glycol-grafted polyethylene glycol.

First, EO was copolymerized with AzPheEO and mPEG-EPO (Scheme 1). For this, TBAB, AzPheEO, and mPEG-EPO were dried under vacuum for 2 h, after which they were exposed to dry nitrogen. Toluene was added to the flask to dissolve mPEG-EPO. Subsequently, the EO solution and i-Bu_3_Al were added to the flask in an ice bath. The solution was stirred for copolymerization at 25 °C in the dark for 18 h. Methanol was added to the solution, and the solvent was removed under reduced pressure. The crude compound was dissolved in acetone, and the solution was dialyzed against water for three days. The resulting polymer was then freeze-dried and is referred to as AzPEGx [y], where x and y represent the Mn and feed ratio of mPEG-EO, respectively. The obtained AzPEGx [y] polymer was characterized via ^1^H-nuclear magnetic resonance (NMR) spectroscopy (JNM-ECZ400R, 400 MHz, JEOL, Tokyo, Japan) in chloroform, as well as via gel permeation chromatography (GPC; JASCO, Tokyo, Japan). The system was equipped with a refractive index detector (RI-2032, JASCO, Tokyo, Japan) and two columns (SB-803 HQ and SB804 HQ; Showa Denko, Tokyo, Japan) in dimethylformamide (DMF) containing 10 mM lithium bromide.

Furthermore, the ultraviolet (UV) absorbance spectra (V−750, JASCO, Tokyo, Japan) of the polymers and 4-azidobenzoic acid were obtained. The polymer and 4-azidobenzoic acid were dissolved in a mixture of ethanol/water (0.1 wt.%). The UV absorbance of each solution was measured between 200 and 400 nm.

### 2.3. Contact Angle Measurements

An unpatterned AzPEGx [y]-coated plate was placed on the stage of a CA−W Automatic Contact Angle Meter (Kyowa Interface Science, Saitama, Japan), and a drop of water (0.1 µL) was placed on the sample surface. The contact angle of the drop with respect to the surface was measured at room temperature. At least 10 angles were measured in different areas, and the results are expressed as the average values with the standard deviation.

### 2.4. Photoimmobilization of AzPEGx [y]

**Scheme 2:**
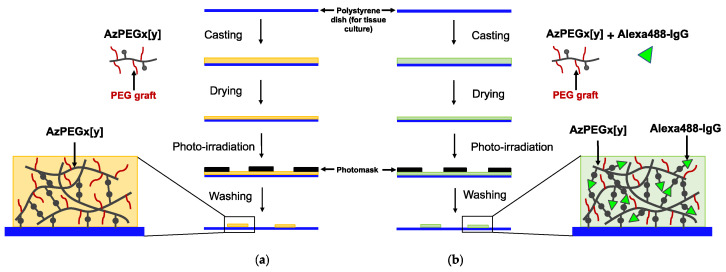
Micropatterning of the prepared AzPEGx [y] polymer (**a**) without and (**b**) with fluorescent-labeled IgG. The circular micropattern was identical to the diameter of the photomask holes.

The photoimmobilization procedure is illustrated in Scheme 2a. The prepared AzPEGx [y] polymer (0.25 wt.% in water, 100 µL) was placed on polystyrene dishes (for tissue culture, Iwaki, Tokyo, Japan). These dishes were then dried at 40 °C under vacuum. A stainless steel punch sheet with 500 μm holes (Yasutoyo Trading, Tokyo, Japan) was placed on the substrate as a photomask, and the surface was exposed to UV light for 20 s using a photoirradiator (45 mW/cm^2^ at 270 nm, L5662 UV spotlight source, Hamamatsu photonics, Hamamatsu, Japan). The surface was then washed with water and subsequently immersed in water for one day. The plates were dried under vacuum and stored in a dark desiccator until further use. To confirm the photoimmobilization of the polymer on polystyrene, the AzPEGx [y] (0.25 wt.%, 90 µL) solution was mixed with Alexa488–IgG (0.25 wt.%, 10 µL) and co-immobilized on the plate (Scheme 2b). Furthermore, fluorescence images were obtained using a fluorescence microscope (Olympus, Tokyo, Japan).

### 2.5. Protein Adsorption

A solution of Alexa488–IgG (0.25 wt.%, 100 µL) was placed onto the AzPEGx [y]-coated surfaces and allowed to stand for 2 h at 25 °C. The substrates were then washed with water and immersed in water overnight. The substrates were then dried under vacuum, and fluorescence images were acquired using a fluorescence microscope (Olympus).

### 2.6. Cell Adhesion

Mouse fibroblast (3T3) cells (Japanese Collection of Research Bioresources, Osaka, Japan) were cultured in Dulbecco’s Modified Eagle’s Medium (Sigma-Aldrich, St. Louis, MO, USA) supplemented with 10% FBS and antibiotics (penicillin (100 units/mL) and streptomycin (100 µg/mL)). The cells were harvested using a 0.25% trypsin–ethylenediaminetetraacetic acid solution. The cell suspension (1.0 × 10^6^ cells) was seeded onto the AzPEGx [y]-coated dishes and incubated at 37 °C in a humidified atmosphere with 5% CO_2_ for 24 h. After incubation, the dishes were washed thrice with phosphate-buffered saline, and the cells were observed using a phase-contrast microscope (Olympus, Tokyo, Japan).

## 3. Results and Discussion

### 3.1. Synthesis of Photoreactive PEG (AzPEGx [y])

EO was copolymerized with AzPheEO and mPEG-EPO via activated ring-opening polymerization. TBAB was used as an initiator and i-Bu_3_Al as the monomer activator to generate polymers with various PEG chain lengths and azidophenyl group contents.

Table 1 summarizes the AzPEGx [y] polymers synthesized via the copolymerization of AzPheEO with EO and two different molecular weights of mPEG-EPO. The azidophenyl group content was confirmed via UV absorbance spectroscopy. The molecular weights and polydispersity indices (PDIs) of the polymers were analyzed using GPC. The AzPheEO and mPEG-EPO contents in the copolymers were found to be lower than those in the feed. The decrease in the ratio of mPEG-EPO was greater than that of AzPheEO. This can be attributed to the high steric hindrance of the PEG moiety in mPEG-EPO. The PDI increased at a lower proportion of the azidophenyl group due to the steric hindrance of mPEG-EPO. In the absence of mPEG-EPO, the polymer did not dissolve in pure water. Therefore, Az [10] was not used for further investigations.

**Figure 1:**
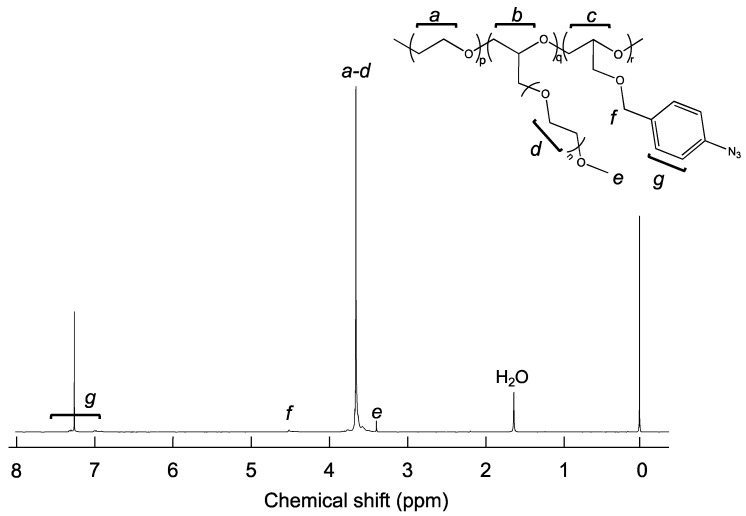
^1^H NMR spectrum of AzPEG750 [5] (solvent: chloroform–d).

The ^1^H NMR spectrum of the synthesized AzPEG750 [5] is presented in Figure 1. Peaks corresponding to the protons of the PEG ethylene group units and methyl protons of the grafted PEG chain were observed at 3.6 and 3.35 ppm, respectively. In addition, peaks corresponding to benzylic protons, aromatic protons, and azidophenyl units were observed at 4.54, 7.37, and 7.05 ppm, respectively.

**Figure 2:**
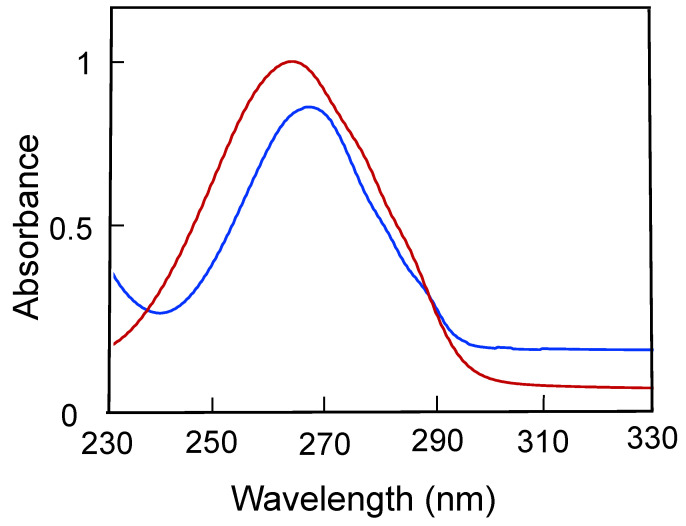
UV absorbance spectra of AzPEG750 [5] (red) and 4-azidobenzoic acid (blue) in ethanol/water (0.1 wt.%).

The UV absorbance spectrum of AzPEG750 [5] and 4-azidobenzoic acid is presented in Figure 2. The peak of the azidophenyl group slightly shifted to a lower wavelength. The azidophenyl group was considered to be in the hydrophobic region of the polymer.

**Figure 3:**
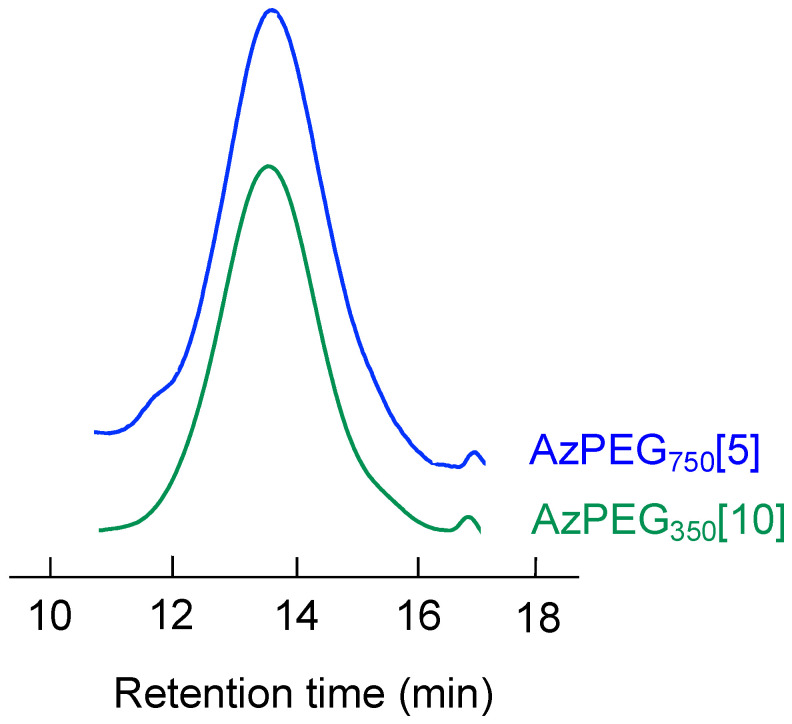
GPC charts of AzPEG350 [10] (green) and AzPEG750 [5] (blue), proving that the azidophenyl group was successfully synthesized via the copolymerization of AzPheEO with EO and mPEG-EPO. Eluent: DMF containing 10 mmol/L LiBr. Flow rate (1 mL/min).

The molecular weight and distribution of AzPEGx [y] were determined via GPC (Figure 3). The prepared AzPEGx [y] polymer appeared as a unimodal trace. This revealed an almost complete polymerization between mPEG-EPO and AzPheEO.

### 3.2. Contact Angle Measurements

Water contact angle measurements were performed to investigate the surface properties of AzPEGx [y] immobilized without a photomask (unpatterned surfaces). As indicated in Table 2, the contact angle on the photoimmobilized surface was lower than that on the non-immobilized surface. This alteration in the contact angle was assumed to be caused by PEG coverage. The hydrophilicity of the immobilized surface was almost independent of the copolymer.

### 3.3. Formation of Micropatterns

**Figure 4:**
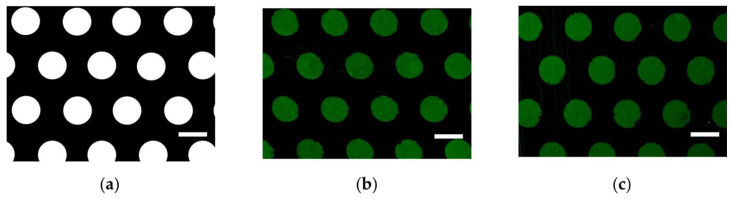
(**a**) Phase-contrast image of photomask. (**b**) Micropatterning test of AzPEG350 [10] and (**c**) micropatterning test of AzPEG750 [5] performed using photomask. The micropattern plate had two different regions, as indicated by the green and dark sections. In the photomask, the green section allowed UV light to enter the AzPEGx [y]–Alexa488IgG complex, resulting in photoimmobilization, which was not possible in the dark sections. Scale bars: 500 μm.

According to Scheme 2b, AzPEGx [y] was micropatterned on the polystyrene surface, as presented in Figure 4. Mixed Alexa488–IgG was co-immobilized with AzPEGx [y] after UV exposure, and the immobilized pattern was the same as that on the photomask. Notably, upon UV irradiation, the highly active nitrenes produced from the azidophenyl moieties randomly attacked the polymer, proteins, and substrate. Consequently, polymer–polymer crosslinks of AzPEGx [y]–AzPEGx [y], AzPEGx [y]–Alexa488–IgG, and AzPEGx [y]–polystyrene substrates were obtained (Scheme 2b).

### 3.4. Protein Adsorption

**Figure 5:**
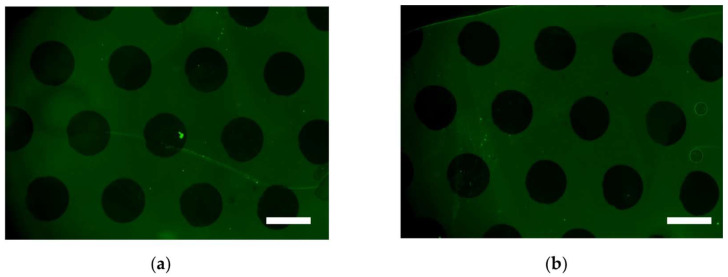
Fluorescence microscopy images of (**a**) AzPEG350 [10]– and (**b**) AzPEG750 [5]–coated micropatterned polystyrene plates after the adsorption of Alexa488-conjugated IgG. Scale bars: 500 μm.

To assess the nonspecific adsorption of proteins on immobilized PEG surfaces, a fluorescent protein (Alexa488-conjugated IgG) was added to the surface prepared based on Scheme 2a, and the resulting structure was examined via fluorescence microscopy (Figure 5). The immobilized AzPEGx [y] suppressed the adsorption of the fluorescent-labeled proteins. The suppression effect did not significantly depend on the nature of AzPEGx [y]. These results revealed that the immobilized surface suppressed nonspecific protein adsorption.

### 3.5. Cell Adhesion

**Figure 6:**
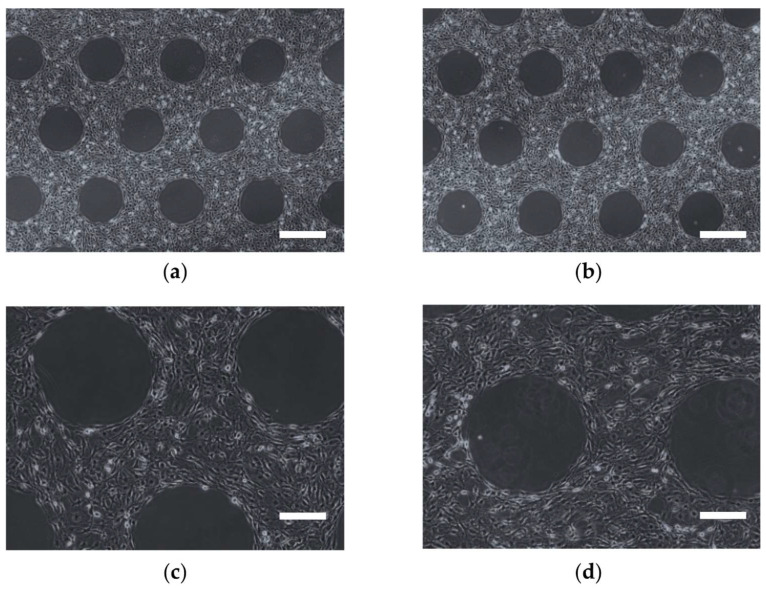
Phase-contrast images of 3T3 mouse fibroblast cells cultured on the AzPEG350 [10]-coated (**a**,**c**) and AzPEG750 [5]-coated (**b**,**d**) micropatterned polystyrene plates at 0.25 wt.%. Scale bars: (**a**,**b**) 500 μm; (**c**,**d**) 200 μm.

Furthermore, cell adhesion onto the AzPEGx [y]-coated polystyrene substrates was evaluated using 3T3 mouse fibroblast cells (Figure 6). For this, 3T3 cells were cultured on the micropatterned surfaces for 24 h. The non-cell-adhered pattern was found to be identical to the hole pattern on the stainless steel photomask. The cells only adhered to the non-UV-exposed areas. The prepared AzPEGx [y] polymer almost completely suppressed cell adhesion due to the introduction of hydrophilic PEG. The suppressive effect on cell adhesion did not depend on AzPEGx [y].

Notably, PEG is a representative antibiofouling polymer. PEG immobilization on biomaterial surfaces is typically conducted for various purposes. Previously, we prepared a PEG-based polymer containing azidophenyl groups in the side chains [21]. However, the polymer was not completely soluble in pure water. Therefore, copolymerization with mPEG-EPO was performed herein, and the resulting copolymer was found to be completely soluble in pure water; it was mixed with a fluorescent protein and immobilized as a mixture on the substrate. The antibiofouling effect was attributed to the hydrophilicity and repulsion of the amphiphilic and flexible grafted polymer chains. Such a copolymer may be useful for micropatterning water-soluble proteins and biomacromolecules with mixing.

Photoreaction is an effective and reasonable method for modifying polymer materials on biomedical devices through covalent binding. The new photoreactive PEG can be coated with water and fixed on the various types of organic substrate by simple photoirradiation. Through the photochemical process, a stable anti-biofouling interface between the polymer and the biomedical devices will be prepared for medical implants, blood contacting tubes, wearable sensors, organ-on-a-chips, microfluidics, and microarray chips.

## 4. Conclusions

In this study, we designed azidophenyl-modified and PEG-grafted PEG (AzPEGx [y]) as photoreactive polymers for surface antibiofouling modifications. Photoreactive polymers were successfully synthesized using AzPheEO copolymerized with mPEG-EPO and EO. AzPEGx [y] significantly reduced the interactions with biological components, such as proteins and cells. Hence, this polymer, with desirable antibiofouling properties, can be used for surface modifications in biomedical applications.

## Figures and Tables

**Table 1:** Characterization of the prepared polymers.

Code	Feed (mol/%)				Composition (mol%) ^a^			Mw ^b^	PDI ^b^
–	AzPhe-EO	EO	mPEG-EPO	mPEG-EPO MW	AzPhe- EO	EO	mPEG- EPO	–	–
Az [10] ^c^	10	90	0	–	9.6	90.4	0	8700	1.6
AzEG350 [10]	10	80	10	350	8.4	85.8	5.8	7800	1.7
AzPEG750 [5]	10	85	5	750	7.4	89.8	2.8	13,000	1.9
AzPEG750 [1]	10	89	1	750	9.9	89.5	0.6	12,000	1.7

^a^ Determined via ^1^H NMR. ^b^ Determined via GPC. ^c^ No PEG-grafted polymer.

**Table 2:** Contact angles on different surfaces.

Surface	Contact Angle (°) ± Standard Deviation
Polystyrene (for tissue culture)	70.7 ± 2.1
Polystyrene with AzPEG350 [10]	41.1± 1.8
Polystyrene with AzPEG750 [5]	41.2 ± 1.6

## Data Availability

All data are available upon reasonable request from the corresponding authors.
